# History and physical exam: a retrospective analysis of a clinical opportunity

**DOI:** 10.1186/s12909-023-04696-1

**Published:** 2023-09-26

**Authors:** David McLinden, Krista Hailstone, Sue Featherston

**Affiliations:** 1 Northern Ontario School of Medicine University, 935 Ramsey Lake Road, Sudbury, ON P3E 2C6 Canada; 2 Huntsville Physicians Local Education Group, 100 Frank Miller Drive, Huntsville, ON P1H 1H7 Canada; 3 Algonquin Family Health Team, Howland Building, 100 Frank Miller Drive, Huntsville, ON P1H 1H7 Canada

**Keywords:** Program implementation, Medical learners, Confidence, Clinical skills, Communication, Feedback

## Abstract

**Background:**

All learners at the Northern Ontario School of Medicine University complete a longitudinal integrated clerkship experience in their third year, which serves to improve learner experience with community and clinical acute and chronic health needs. Early in the program, Muskoka faculty (two of the 15 LIC sites of NOSM U) became aware that learners never had the occasion to complete a full history and physical exam on a real patient with complex needs. Recognizing this as a critical experience, a program was initiated to provide learners with this opportunity. This manuscript reports on the effectiveness and impact of this novel program and outlines the procedure developed to incorporate a similar program should communities see the relevance.

**Methods:**

Using a mixed method design, feedback was collected from learners and preceptors following the implementation of a novel learning opportunity for clinical clerks. Learners completed a full history and physical exam on volunteer complex patients, with supervision and immediate feedback. Using semi structured surveys, data was collected from each learner and preceptor to determine the program impact and optimize the program. Laurentian University research ethics board, certificate number 6021120.

**Results:**

Both learners and preceptors agreed this was a valuable experience for learners, a good use of their time and contributed to essential skills including, communication, time management and appropriate data collection. The use of real patients was reported to be very appropriate by learners and faculty and often highlighted gaps in the learner’s knowledge that they were then able to address.

**Conclusion:**

Feedback collected in this study confirms that providing medical learners the opportunity to complete a full history and physical exam with supervision and feedback was significantly beneficial from both a clinical and a skills-based aspect. Requiring learners to complete this task within the established period forced them to manage their time, focus on clinical consideration and remain on task. Enhancing learning opportunities is associated with improved outcomes and understanding in medical learners. Positive community experience is also related to learner retention, which is paramount for attracting new physicians in a time with significantly limited human health resources.

**Supplementary Information:**

The online version contains supplementary material available at 10.1186/s12909-023-04696-1.

## Background

Longitudinal Integrated Clerkship (LIC) is increasingly being integrated in medical education curriculum acknowledging the importance front-line experience provides to medical trainees [1–3]. Real-world experience in primary and community care settings has been found to result in positive learning outcomes for learners, while laying the solid foundation for comprehensive and compassionate care [1–3]. Additionally, exposure to general practice and these role models earlier in a learner’s education has been found to positively influence their choice to specialize in general practice [4].

LIC placements usually occur during the year three or four of undergraduate medical education and are designed to deliver learners the opportunity to practice medicine in a functional clinical environment [3, 5]. These clerkships are referred to as ‘integrated’ because they involve continuous and concurrent community and hospital based clinical experiences for each learner [3]. This long-term experience allows learners to become immersed in the community’s well-being, acute and chronic health needs [3]. While LICs are intended to provide a full scope of experiences for the learners, gaps in their learning do occur related to the LIC site, the available focused learning opportunities, and the lack of formal structure associated with each placement. For medical schools such as Northern Ontario School of Medicine University (NOSMU), this community experience is crucial for upholding a model of social accountability, by providing learners firsthand experience of working to improve the health of the people and communities of Northern Ontario [6].

It is widely agreed that when learning to practice medicine, there is no replacement for the authentic opportunity to learn from real patients [7, 8]. Early contact with patients has shown to enhance learner motivation, teach them things that ‘cannot be learned from books,’ and increase their confidence when interacting with patients [9]. Working alongside clinicians in their practice provides learners the experience and skill development for clinical reasoning, communication, history taking, and physical examination [9]. Importantly, studies have found that experiential methods such as LICs are more effective in teaching communication skills than traditional lectures, in part due to the immediate feedback provided by preceptors in a clinical setting [9, 10].

NOSMU (formerly known as NOSM) is a medical school with primary campuses in Sudbury and Thunder Bay but its reach encompasses most communities of Northern Ontario and it is the first independent medical school in Canada. The school has incorporated LIC into their curriculum which all learners participate in during year three (LIC Report, 2020). In fact, NOSMU was the first medical school in the world in which all learners are required to complete a LIC as part of their educational obligations [6].

Prior to the educational opportunity described herein, NOSMU learners performed aspects of history taking and physical exams, however, were without the occasion to conduct a full history and physician exam on an actual complex patient. Simulated patients are often incorporated in experiential learning and while they can be a valuable teaching tool, they often fail to express the wide range of clinical signs and symptoms that can only be appreciated from an examination on a real patient [11]. Additionally, the requirement to deduce clinical details while staying on-topic is a far more practiced skill with actual patients than can be demonstrated with simulated ones [11].

NOSMU offers LIC placements in 15 communities in Northern Ontario, including the towns of Huntsville and Bracebridge, where learners have been hosted since 2007 [6, 12]. Huntsville and Bracebridge are small, rural communities located approximately two hundred and thirty kilometers north of Toronto, spanning significant geography within Muskoka Ontario and home to approximately 15,000–20,000 permanent residents in each community [13]. Seasonal tourism increases the populations in these communities several-fold, thus creating dynamic and unique healthcare needs. This mixed methods study investigates an innovative learning approach that is currently only offered in Huntsville and Bracebridge (Muskoka), two sites of the NOSMU LICs.

After hosting the initial NOSMU LIC learners, preceptors noted that while learners were provided many opportunities for focused learning (emergency department, specialty clinics, and very busy family practices) at no time did they have the opportunity to complete a full history and physical exam independent of their preceptor. Further inquiry confirmed that learners only completed this essential skill with simulated patients, thus lacking the appropriate communication and prioritization clinically required with complex patients [11]. Current literature highlights the need for and importance of clinical experiences in medical education, while also noting that significant gaps remain [14, 15].

Collecting an accurate history and physical exam is a critical diagnostic tool, particularly for new physicians [11]. Recognizing this crucial step in physician training, the Huntsville Physicians Local Education Group supported a pilot project providing third year learners from NOSMU the opportunity to complete full history and physical exams on complex volunteer patients. The process was supervised at all times and clerks were provided valuable feedback, which is foundational in optimizing clinical skills [16]. Preceptor and learner feedback has been collected after each session and reviewed annually. Based on feedback, modifications have been made to improve the learner and preceptor experience.

In rural, underserved communities such as Huntsville and Bracebridge, positive LIC experiences are crucial, as they can lead to enhanced recruitment of new physicians (LIC Report, 2020). In fact, positive clinical and educational experiences in rural settings are significantly associated with learners’ choosing to return to establish their career [5]. LIC provides continuity of preceptorship and establishes better learner-teacher relationships, allowing the educational experience to be more tailored to individual learner’s needs (LIC Report, 2020). Barriers do exist with LIC and may be site-related including curriculum design, lack of required infrastructure, limited preceptor capacity, learner preference, and community capacity (LIC Report, 2020).

The initial hypothesis in designing this program was that providing LIC learners with the opportunity to complete a full history and physical exam with feedback would enhance their organization, confidence, and communication skills. Equally important was determining the impact of this program on local preceptors with respect to their time, satisfaction, and supervisory experience. Review and discussion of the findings indicate that this program may be relevant in other similar sized communities, both within NOSMU and for other medical schools. Significant details are included so that other communities who may see the significance of this enhanced learning opportunity can incorporate it in their LIC rotations. Data collected from the learners and preceptors over several years has been collated and analyzed for statistical relevance and qualitative themes.

## Methods

We have followed the SRQR and STROBE Cohort guidelines, including information within the manuscript on: background, survey development, methodology (including sampling rationale), analytic approach and discussion (including limitations). We have utilized guidelines for reporting observational studies from the Strengthening the Reporting of Observational Studies in Epidemiology (STROBE) statement for surveys analysis and followed the Standards for reporting qualitative research (SRQR) checklist during manuscript preparation [17, 18].

The data included in this report includes all collected survey results between September 2016 to April 2020. Providing feedback by preceptors and learners was voluntary and while most participants completed them, not all questions were answered by all participants resulting in slight discrepancy of total responses. The qualitative approach is grounded theory reporting on both learner and preceptor perspective. Quantitative responses were collated and analysed using Microsoft Excel (2022). Data analyses included descriptive statistics. Missing data were not imputed. Qualitative responses were reported from both learners and preceptors and common themes were identified.

 Participants included all LIC learners in Huntsville and Bracebridge (South Muskoka site). The first iteration of the program was voluntary with 100% of participants [7] taking part. In subsequent years participation was mandatory, feedback was voluntary. In total 31 learners were included and 16 preceptors. Preceptors were NOSMU faculty from each community (Huntsville and Bracebridge) and were almost exclusively family practitioners or emergency physicians. The principal investigator was not scheduled in the learner supervision.

The program was hosted within the NOSMU classroom at the Huntsville site of Muskoka Algonquin Healthcare. The site liaison coordinator scheduled preceptors, learners, and volunteer complex patients (scheduling template found in Additional file 1). Preceptors were compensated for their time; volunteers were provided a stipend for their participation. Surveys were distributed to the preceptors and the learners by the SLC at the start of the day and were submitted anonymously when the session is complete (Additional files 2 and 3). Surveys included both quantitative and qualitative responses, which were analysed accordingly.

The program initially ran three times per academic year, which was reported to be too onerous on learners and preceptors, after which it was reduced to twice per year, scheduled at the start and the mid-point of the clerkship rotation (September and January). Learners completed a full history and physical exam on a complex volunteer patient under direct supervision by the preceptor. Throughout the years a database of “legacy patients” who regularly participate and enjoy their involvement has been established. Patients were counselled, given the opportunity to ask questions, and provided a handout detailing the program (Additional file 4). Learners were divided into two groups, one group completing physical exams and histories on the volunteers, while the other group observes one of their peers via audio-visual monitoring. Following the experience, they switch roles ensuring every learner has opportunity to participate.

The scheduled protocol includes a fifteen-minute introduction of the preceptor to the patient. The preceptor then introduces the learner and explains the procedure for the student to complete a history and exam then review their findings with the preceptor. The learner then has forty-five minutes to complete a full history and physical exam on the patient, with the preceptor remaining in the room as an observer. Learners are prompted 10 min prior to the end of the exam. Preceptors were able to choose, at their own discretion, to provide real-time feedback to learners or to take on an observational role. Following the exam, learners have ten minutes to gather their thoughts, after which they provide a five-minute case presentation to their preceptor followed by a debrief with them for thirty minutes. This is repeated with the same patient, preceptor and a second learner before the patient is provided a stipend for their time.

Originally, schedules for this experience were arranged so that each group of learners completed the entire activity, including debrief and case discussion, before the next group began. However, for efficiency this was changed to a rotating schedule where there were continuous physical exams and histories with the different learners (Additional file 1). When coordinating this schedule, it was important to confirm that learners were paired with a preceptor who was not supervising their clerkship to provide learners novel and unbiased feedback. This schedule was established having access to two exam rooms and a meeting room. One exam room was a designated teaching space with the audiovisual connection to the meeting room facilitating observation of skills/procedures.

In the initial years, learners entered the activity directly without any prior discussion with a preceptor. However, based on observation and feedback a thirty-minute didactic session with the site liaison coordinator was implemented to outline learners’ expectations and to enhance their learning experience.

At the end of each patient history and physical exam, each learner and preceptor were invited to complete the feedback survey to evaluate the project (Additional files 2 and 3). The feedback collected over the last four year period has been collated and analyzed in this report to determine if the process is helpful, an appropriate length, a valuable learning tool, appropriate for the academic level, etc. Respondents were invited to evaluate the process using a Likert scale and qualitative open-ended questions. Responses were anonymized with no participant information collected. This feedback survey was used throughout the study to modify and improve the program to maximize its positive impact on medical learners’ clinical education. The program feedback collected and presented here is not in any way used for learner evaluation. A confidential evaluative aspect of learner performance is included in their overall LIC evaluation but is not linked to any feedback survey results (Additional file 5).

This study has received ethical approval through the Laurentian University research ethics board, certificate number 6,021,120.

## Results

Triangulation was used in this study to enhance the richness of the qualitative data and to increase the validity and reliability of results [19].

Preceptors and learners responded to the set of questions found in figure one and two respectively. The responses used a Likert scale ranging from one to ten, with one being ‘Strongly Disagree’ and ten being ‘Strongly Agree’. Both learners and preceptors were also given the opportunity in the survey to provide open-ended semi-structured feedback on each question and suggestions for general changes to the program.

### Preceptors and learners generally felt that the time allotted for the history and physical exam was appropriate and learners confirm it was a valuable experience that should be routinely included in LIC

**Fig. 1:**
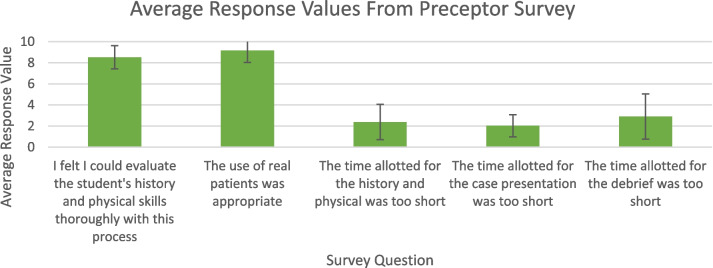
Average response values from preceptor survey show that they generally agree that this experience was implemented appropriately

**Fig. 2:**
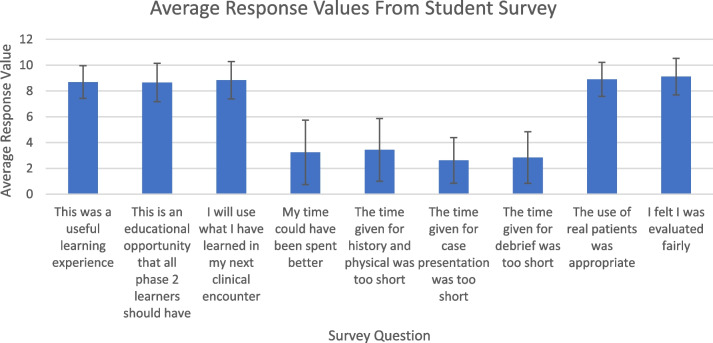
Average response values from student survey show a positive perception of this experience

Preceptors and learners indicate that the time provided was adequate and appropriate (Figs. 1 and 2). Some preceptors noted however, that 45 min would not be practical in a clinic situation but confirmed it was optimal for learners. Several learners indicated time management was a challenge for the full exam, particularly with complex patients. The opportunity to improve time management skills throughout this opportunity was noted. Learners appreciated the use of prompts, especially in their first exam session. Additionally, learners noted that these sessions allowed them to be *“more focused and deliberate about my history telling and physical exam skills”.*

### Learners support this opportunity, agree this is a useful learning experience, their time was well spent, and they will apply knowledge learned in their next clinical encounter

Learners overwhelmingly agree that this was a valuable opportunity that all LIC learners should have, and their comments underscore the value of working with real and complex patients to support learning appropriate data collection and time management skills (Fig. 2). *“Really allows us to find gaps in our full history and physical when it is observed by a clinician”.* It was noted that this opportunity highlighted pedagogical areas that needed more focus, and allowed them to practice and expand on their skills in a low-pressure environment. Learners appreciated the direct observation and feedback of their clinical skills informing them about specific areas to work on throughout their clerkship. Repeating the process provided learners with the opportunity to affirm their skill development over time. It was specifically noted that completing this history and physical exam opportunity early in their clerkship allowed them to incorporate the skills learned throughout their placement *“Feedback received will be definitely incorporated into my next encounter”*.

### Preceptors and learners generally agree that the use of real patients is appropriate

Preceptors and learners almost exclusively agree that the use of real complex patients was appropriate for this opportunity (Figs. 1 and 2). Real patients allow the experience to be more relevant and representative of “real life experience.” It was noted that real patients, particularly those with complex issues require better time management, which is a learned skill. Learners, in some cases spent too long on gathering patient history, leaving less time for a full exam. Suggestions were made by learners to counsel patients prior to provide more focused discussion, and learners reported that it might be helpful to have patients report only one specific complaint. Others highlighted the need to learn management of complex patients within a specified time, particularly when the presenting issue is not clear, which is more closely representative of actual practice. *“It can be a nice challenge to see patients with multiple co-morbidities and concerns”.*

### Preceptors were able to evaluate the learner’s skills related to the task and learners agree that these evaluations were fair, constructive, and helpful

Preceptors agreed that they were able to fairly evaluate learners and learners felt that they were fairly evaluated (Figs. 1 and 2). Responses and personal feedback were reported to be effective, helpful, and appreciated in strengthening these essential skills. Learners reported appreciation for constructive criticism and reinforcement of skills they are required to use on a regular basis; *“Preceptor feedback was critical but appropriate”.*

## Discussion

This study is intended to report on the feasibility and appropriateness of a novel opportunity for third-year medical learners completing their LIC at NOSMU. LIC is an experience meant to provide medical learners with hands on clinical experience that will further develop their skills, expertise, and highlight areas for improvement before residency. NOSM U is the most recently established and only stand-alone medical school in Canada. A priority for the university includes training rural residents who are socially accountable in their career. Training undergraduate learners in rural locations can be challenging as their experience is directly related to the health issues and conditions of their community. Early observations by NOSMU rural faculty in Huntsville and Bracebridge found that learners were without the critical experience of managing a complex patient to obtain a full history and physical exam. Regardless of where undergraduate learners choose to practice or what specialty they select, successful completion of a formal exam is imperative in their ability to provide quality patient care.

Observation and direct feedback or debriefing are linked with improved results in medical education. In fact, most graduate and medical schools have transitioned to competency-based training [20]. Competency based learning encourages students to continue to expand their skill set as they incorporate the previously learned skills in the daily practice. Optimally, feedback should occur immediately after a skill is practiced and be communicated directly by the observer [21]. This type of learning can occur over the span of a professional career developing into a coaching relationship [20, 21].

The goal of this study was to evaluate the effectiveness of a program that provides a clinical skill to undergraduate medical learners with observation and immediate feedback. Based on the feedback collected throughout the program both learners and preceptors confirmed that this clinical opportunity was a positive and beneficial learning experience. Direct observation and immediate feedback is a valuable, underutilized tool in medical education that provides learners with confidence, communication sills, immediate learning and knowledge retention [22]. The feedback collected with respect to time allotment, skills training, frequency of the activity, and inclusion of real complex patients confirm this activity is both feasible and valuable. Learners consistently reported that the honest and immediate feedback was vital in allowing them to modify and improve their approach when taking patient histories and competing a physical exam.

Preceptors were highly engaged in this activity with many returning annually to participate in this program. Preceptors appreciated the opportunity to observe the learners at an early stage in their LIC and noted what a great, and rare, opportunity it was to observe a learner conducting a complete exam. While the complex real patient was reported to be difficult by some learners, preceptors reported their role was “essential” and allowed learners to practice “patient centered interview skills” while learning techniques to remain focused on what is medically relevant.

### Timing of activity and use of real patients was appropriate

Both learners and preceptors felt that the structure of the activity was appropriate. They indicated that the timing allotted for the physical exam and history, case presentation, and debrief was suitable for the learning context of this experience. Preceptors and learners discussed the importance of learning time management during this portion of the exercise, as this skill is essential for learners to have during both their clerkship and future practice. The use of real complex patients was found to be both beneficial and problematic by preceptors and learners. Both groups appreciated the actual history and exam on complex patients, more so from the preceptors. A few learners and preceptors mentioned that having one complaint to address may have resulted in a more seamless process. However, this speaks to the initial objective of the study to develop the skills required to ensure all aspects of the exam were complete while remaining focused on the medically relevant issues. Standardized patients with one primary concern are seldom reality in practice.

Effective and efficient communication with patients having complex medical concerns is an essential skill for learners to develop and is seldom provided in undergraduate medical environments. This interaction helps them learn to recognize and decipher important medical information while remaining on task and within time constraints. Communicating versus conversation with real patients, where redirection to medical concerns may be required, is a skill physicians need to develop to provide optimal care in a timely manner. The opportunity to provide this direct, individual, immediate feedback where learners can discuss the findings and inquire about areas they can improve and techniques that work for others is invaluable in developing a trusting environment that facilitates learner success [23].

### Changes made to the experience implementation were met with positive feedback

Establishing an open line of communication and feedback from the start of the LIC demonstrates a commitment to the learners. Based on their feedback early changes to the program were made to ensure there were not excessive time commitments being imposed without significant clinical and academic value. The shift in this program to twice per year at times without significant competing priorities was critical in its success. Adding a didactic session prior to the exercise outlining procedures, expectations, components of the full exam, and an opportunity for questions was also well received. Positive feedback from all parties has been routinely collected since these minor modifications. Implementing this type of structured program requires continual feedback that can be acted on to maintain positive learning opportunities and keep faculty engaged and committed. Minor adjustments such as upgrading interview offices to fully functional clinical spaces was a small change that was appreciated by learners.

### Preceptor engagement and feedback is essential to learner learning in the clinical environment

In terms of faculty engagement, it was clear that preceptors genuinely enjoyed and appreciated the opportunity to supervise learners completing this activity. Faculty engagement is critical in positive academic communities. It encourages faculty to remain current, engaged in their specialty and teaching practices, and provides personal and professional satisfaction. Teaching clinical skills to undergraduate learners can impose significant time restraints on faculty, particularly in rural communities who are often without protected time. Ensuring that these additional sessions are valuable by collecting and sharing feedback from all parties is positively associated with increased participation. Faculty need to know their commitment is relevant, appreciated and utilized by learners to enhance their medical learning [24].

Positive experiences also provide learners more satisfaction and can result in improved learner retention and potential recruitment, which it crucial in the current climate of provider shortages. In this program learners were provided the appropriate didactic material prior to the session then allowed to observe their peers conducting the exercise. Peer feedback and discussion with respect to clinical skills reinforces their professionalism, teamwork and collegial communication skills, particularly when preceptors are present [25, 26]. Completing this activity twice annually provides learners the opportunity to witness their growth and maturity, along with their colleagues’. Self-assessment and that of peers is an important step in continued education [27]. The opportunity to identify ‘gaps’ in learners’ and hone skills early in the LIC to practice those skills throughout the year was noted by many.

## Conclusion

LIC provides learners an opportunity to become immersed in the community they are placed in while learning not only clinical skills but also communication, collegiality, time management, prioritization, and organization, both personally and professionally. Host medical communities vary by their patient population, available services, and in the provision of specialized skills clinics for learners. Huntsville and Bracebridge consistently offer a wide variety of clinics to enhance learner skills in; suturing, musculoskeletal conditions, cardiac care, pediatrics to name a few. For each clinical experience the ability to complete a full history and physical exam is imperative, which was notably absent for actual patients in undergraduate training. This program aimed to investigate the feasibility and impact of offering what has become a full day of clinical training for LIC learners on appropriate history and physical exams. The findings confirm this is valuable experience for both the learners and the faculty. This activity has gained substantial popularity within the undergraduate learner community and incoming LIC students look forward to the opportunity. Providing learners with the occasion to address skills that need development early in their clerkship encourages introspection and highlights the value of peer and collegial support, feedback, and collaboration.

### Supplementary Information

**Additional file 1.**

**Additional file 2.**

**Additional file 3.**

**Additional file 4.**

**Additional file 5.**

## Data Availability

Data for survey results (quantitative and qualitative) are available upon request. Additionally, example of documents required to establish this type of program are included in the manuscript, such as: Example of Patient Handout, Example of Rotating Schedule (*N* = 7 learners, *N* = 4 preceptors, and *N* = 4 patients), Example of Learner Survey, Example of Preceptor Survey, Example of learner evaluation sheet. No learner evaluations are reported. Requests for data should be directed to Krista Hailstone at krista.hailstone@mahc.ca.
