# Supplementary material for: History and physical exam: a retrospective analysis of a clinical opportunity
Source: BMC Med Educ. 2023 Sep 26;23:699. doi: 10.1186/s12909-023-04696-1 (PMC10523620; doi:10.1186/s12909-023-04696-1)
Supplement: Supplementary file 1 — Additional file 1. [file 12909_2023_4696_MOESM1_ESM.docx]

**Additional file 1**

**Example Template Schedule: Complete Observed History and Physical Exam Session**

| **Time** | **What** | **Who** | **Where** |  |
| --- | --- | --- | --- | --- |
| 11:15-11:45 | Didactic Session | Preceptor 4 and all clerks |  |  |
| 11:45-12:15 | Meet Site Liaison Coordinator (SLC) | SLC and all clerks |  |  |
| 11:45-12:00 | Patient Orientation | Preceptor 4 and patients 1&2 |  |  |
| 12:00-12:15 | Preceptor 1 meets patient 1 | Preceptor 1 and patient 1 |  | 12:00 – Patients 1&2 and Preceptors 1&2 arrive  12:15 – Students 1&2 start; the rest have lunch |
|  | Preceptor 2 meets patient 2 | Preceptor 2 and patient 2 |  |  |
| 12:15-1:00 | Student 1 History and Physical | Student 1, preceptor 1, patient 1 |  |  |
|  | Student 2 History and Physical | Student 2, preceptor 2, patient 2 |  |  |
| 12:30-12:45 | Patient orientation | Preceptor 4 and patients 3&4 |  | 12:45 – Patients 3& 4 Arrive |
| 12:45-1:00 | Preceptor 3 meets patient 3 | Preceptor 3 and patient 3 |  |  |
|  | Preceptor 4 meets patient 4 | Preceptor 4 and patient 4 |  |  |
| 1:00-1:45 | Student 1 case presentation and feedback | Student 1, preceptor 1, patient 1 |  |  |
|  | Student 2 case presentation and feedback | Student 2, preceptor 2, patient 2 |  |  |
|  | Student 3 History and Physical | Student 3, preceptor 3, patient 3 |  |  |
|  | Student 4 History and Physical | Student 4, preceptor 4, patient 4 |  |  |
| 1:45-2:30 | Student 3 case presentation and feedback | Student 3, preceptor 3, patient 3 |  |  |
|  | Student 4 case presentation and feedback | Student 4, preceptor 4, patient 4 |  | ~2:00 – Patient 4 goes home after case presentation |
|  | Student 5 History and Physical | Student 5, preceptor 1, patient 1 |  |  |
|  | Student 6 History and Physical | Student 6, preceptor 2, patient 2 |  |  |
| 2:30-3:15 | Student 7 History and Physical | Student 7, preceptor 3, patient 3 |  |  |
|  | Student 5 case presentation and feedback | Student 5, preceptor 1, patient 1 |  | ~2:45 – Patients 1&2 go home after case presentation  3:15 – Preceptors 1&2 go home  ~3:30 – Patient 3 goes home after case presentation |
|  | Student 6 case presentation and feedback | Student 6, preceptor 2, patient 2 |  |  |
| 3:15-4:00 | Student 7 case presentation and feedback | Student 7, preceptor 3, patient 3 |  |  |

**Example Template for Booking Times**

| Preceptor 4 | 11:15-4:00 |
| --- | --- |
| Preceptor 3 | 11:45-4:00 |
| Patients 1&2 | 11:45-2:45 |
| Preceptors 1&2 | 12:00-3:15 |
| Patient 3 | 12:30-3:30 |
| Patient 4 | 12:30-2:00 |

The schedule is based on the availability of one boardroom room and two exam room spaces. Additional rooms would provide easier scheduling
