# Supplementary material for: History and physical exam: a retrospective analysis of a clinical opportunity
Source: BMC Med Educ. 2023 Sep 26;23:699. doi: 10.1186/s12909-023-04696-1 (PMC10523620; doi:10.1186/s12909-023-04696-1)
Supplement: Supplementary file 2 — Additional file 2. [file 12909_2023_4696_MOESM2_ESM.docx]

**Additional file 2**

**Preceptor Survey Template**

For each of the questions below, please circle the response that best characterizes how you feel about the statement, where 1 = Strongly Disagree and 10 = Strongly Agree.

|  | **Strongly Disagree** |  |  |  |  |  |  |  |  | **Strongly Agree** |
| --- | --- | --- | --- | --- | --- | --- | --- | --- | --- | --- |
| 1.) I felt I could evaluate the student's history and physical skills thoroughly with this process | 1 | 2 | 3 | 4 | 5 | 6 | 7 | 8 | 9 | 10 |
| Additional Comments: | | | | | | | | | | |
|  | **Strongly Disagree** |  |  |  |  |  |  |  |  | **Strongly Agree** |
| 2.) The use of real patients was appropriate | 1 | 2 | 3 | 4 | 5 | 6 | 7 | 8 | 9 | 10 |
| Additional Comments: | | | | | | | | | | |
|  | **Strongly Disagree** |  |  |  |  |  |  |  |  | **Strongly Agree** |
| 3.) The time allotted for the history and physical was too short | 1 | 2 | 3 | 4 | 5 | 6 | 7 | 8 | 9 | 10 |
| Additional Comments: | | | | | | | | | | |
|  | **Strongly Disagree** |  |  |  |  |  |  |  |  | **Strongly Agree** |
| 4.) The time allotted for the case presentation was too short | 1 | 2 | 3 | 4 | 5 | 6 | 7 | 8 | 9 | 10 |
| Additional Comments: | | | | | | | | | | |
|  | **Strongly Disagree** |  |  |  |  |  |  |  |  | **Strongly Agree** |
| 5.) The time allotted for the debrief was too short | 1 | 2 | 3 | 4 | 5 | 6 | 7 | 8 | 9 | 10 |
| Additional Comments: | | | | | | | | | | |

**Suggestions for Change:**
