# Supplementary material for: History and physical exam: a retrospective analysis of a clinical opportunity
Source: BMC Med Educ. 2023 Sep 26;23:699. doi: 10.1186/s12909-023-04696-1 (PMC10523620; doi:10.1186/s12909-023-04696-1)
Supplement: Supplementary file 3 — Additional file 3. [file 12909_2023_4696_MOESM3_ESM.docx]

**Additional file 3**

**Student Survey Template**

For each of the questions below, please circle the response that best characterizes how you feel about the statement, where 1 = Strongly Disagree and 10 = Strongly Agree.

|  | **Strongly Disagree** |  |  |  |  |  |  |  |  | **Strongly Agree** |
| --- | --- | --- | --- | --- | --- | --- | --- | --- | --- | --- |
| 1.) This was a useful learning experience | 1 | 2 | 3 | 4 | 5 | 6 | 7 | 8 | 9 | 10 |
| Additional Comments: | | | | | | | | | | |
|  | **Strongly Disagree** |  |  |  |  |  |  |  |  | **Strongly Agree** |
| 2.) This is an educational opportunity that all phase 2 learners should have | 1 | 2 | 3 | 4 | 5 | 6 | 7 | 8 | 9 | 10 |
| Additional Comments: | | | | | | | | | | |
|  | **Strongly Disagree** |  |  |  |  |  |  |  |  | **Strongly Agree** |
| 3.) I will use what I have learned in my next clinical encounter | 1 | 2 | 3 | 4 | 5 | 6 | 7 | 8 | 9 | 10 |
| Additional Comments: | | | | | | | | | | |
|  | **Strongly Disagree** |  |  |  |  |  |  |  |  | **Strongly Agree** |
| 4.) My time could have been spent better | 1 | 2 | 3 | 4 | 5 | 6 | 7 | 8 | 9 | 10 |
| Additional Comments: | | | | | | | | | | |
|  | **Strongly Disagree** |  |  |  |  |  |  |  |  | **Strongly Agree** |
| 5.) The time given for history and physical was too short | 1 | 2 | 3 | 4 | 5 | 6 | 7 | 8 | 9 | 10 |
| Additional Comments: | | | | | | | | | | |
|  | **Strongly Disagree** |  |  |  |  |  |  |  |  | **Strongly Agree** |
| 6.) The time given for case presentation was too short | 1 | 2 | 3 | 4 | 5 | 6 | 7 | 8 | 9 | 10 |
| Additional Comments: | | | | | | | | | | |
|  | **Strongly Disagree** |  |  |  |  |  |  |  |  | **Strongly Agree** |
| 7.) The time given for debrief was too short | 1 | 2 | 3 | 4 | 5 | 6 | 7 | 8 | 9 | 10 |
| Additional Comments: | | | | | | | | | | |
|  | **Strongly Disagree** |  |  |  |  |  |  |  |  | **Strongly Agree** |
| 8.) The use of real patients was appropriate | 1 | 2 | 3 | 4 | 5 | 6 | 7 | 8 | 9 | 10 |
| Additional Comments: | | | | | | | | | | |
|  | **Strongly Disagree** |  |  |  |  |  |  |  |  | **Strongly Agree** |
| 9.) I felt I was evaluated fairly | 1 | 2 | 3 | 4 | 5 | 6 | 7 | 8 | 9 | 10 |
| Additional Comments: | | | | | | | | | | |

**Suggestions for Change:**
