# Supplementary material for: History and physical exam: a retrospective analysis of a clinical opportunity
Source: BMC Med Educ. 2023 Sep 26;23:699. doi: 10.1186/s12909-023-04696-1 (PMC10523620; doi:10.1186/s12909-023-04696-1)
Supplement: Supplementary file 4 — Additional file 4. [file 12909_2023_4696_MOESM4_ESM.docx]

**Additional file 4**

**[Template] Introduction for Volunteer Patients of an**

**Observed History and Physical Session**

To begin with, thank you very much for doing this. Your participation is essential for the development of these new health professionals as they learn the art and science of becoming a physician.

**What we need from you:**

- A reason why you are visiting the doctor (i.e.: follow up from a test, a new symptom like shortness of breath, belly pain, chest pain, etc.). Please treat this interview like any other visit to a doctor; it will make it more realistic for the student.

**What to expect:**

- Just like your doctor would, the student will ask you why you are here. The student will also ask you many questions about your past medical history. Answer as many as you are able to and comfortable with. You don’t have to hide anything, just answer the questions truthfully. The goal of this exercise is to allow the student to gather and organize real information from a real patient.
- The student will examine you as well. Again, please respond realistically to any exam that the student may do on you.
- A licensed physician will observe the entire session and take notes on the student’s performance so that the student has detailed feedback about their strengths and potential areas of improvement.

As with any visit to a healthcare provider, all information shared is confidential. At any time, if you are uncomfortable with any questioning or examination, please tell the student and the examiner in the room.

There will be a few minutes before we begin when you can ask me any questions you may have. Again, thank you for your participation.

Sincerely,

[NAME AND SIGNATURE OF SUPERVISING PHYSICIAN]
