# Supplementary material for: History and physical exam: a retrospective analysis of a clinical opportunity
Source: BMC Med Educ. 2023 Sep 26;23:699. doi: 10.1186/s12909-023-04696-1 (PMC10523620; doi:10.1186/s12909-023-04696-1)
Supplement: Supplementary file 5 — Additional file 5. [file 12909_2023_4696_MOESM5_ESM.docx]

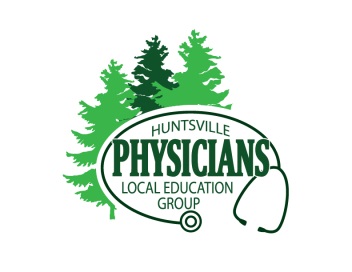
**Additional file 5**

**Observed History and Physical - Evaluation of Students**

Please take a few minutes to fill out this survey on the quality of the service our students provided. We appreciate your feedback and all answers will be kept confidential. Thank you for your participation.

Student Name ______________________

Preceptor Name _____________________

A. MEDICAL EXPERTISE

1. Student possessed appropriate knowledge to fully undertake history and physical.

Strongly Disagree Strongly Agree

1 2 3 4 5 6 7 8 9 10

Comments:

____________________________________________________________________________________________________________________________________________________________________________________________________________________________________________________________________________________________________________________________________________________

2. Student was able to gather data in an organized manner.

Strongly Disagree Strongly Agree

1 2 3 4 5 6 7 8 9 10

Comments:

____________________________________________________________________________________________________________________________________________________________________________________________________________________________________________________________________________________________________________________________________________________

3. Student possessed appropriate exam skills to fully undertake physical exam.

Strongly Disagree Strongly Agree

1 2 3 4 5 6 7 8 9 10

Comments:

____________________________________________________________________________________________________________________________________________________________________________________________________________________________________________________________________________________________________________________________________________________

4. Student demonstrated appropriate problem solving ability through history and physical exam.

Strongly Disagree Strongly Agree

1 2 3 4 5 6 7 8 9 10

Comments:

____________________________________________________________________________________________________________________________________________________________________________________________________________________________________________________________________________________________________________________________________________________

B. COMMUNICATION

1. Student was empathetic and respectful during history and physical.

Strongly Disagree Strongly Agree

1 2 3 4 5 6 7 8 9 10

Comments:

____________________________________________________________________________________________________________________________________________________________________________________________________________________________________________________________________________________________________________________________________________________

2. Student was clear with questions and instructions with patients.

Strongly Disagree Strongly Agree

1 2 3 4 5 6 7 8 9 10

Comments:

____________________________________________________________________________________________________________________________________________________________________________________________________________________________________________________________________________________________________________________________________________________

3. The case presentation was organized.

Strongly Disagree Strongly Agree

1 2 3 4 5 6 7 8 9 10

Comments:

____________________________________________________________________________________________________________________________________________________________________________________________________________________________________________________________________________________________________________________________________________________

4. The case presentation was appropriate for the presenting problem.

Strongly Disagree Strongly Agree

1 2 3 4 5 6 7 8 9 10

Comments:

____________________________________________________________________________________________________________________________________________________________________________________________________________________________________________________________________________________________________________________________________________________

C. TIME MANAGEMENT

1. The student was able to prioritize data gathering and completed the physical exam appropriate to the clinical presentation.

Strongly Disagree Strongly Agree

1 2 3 4 5 6 7 8 9 10

Comments:

____________________________________________________________________________________________________________________________________________________________________________________________________________________________________________________________________________________________________________________________________________________

2. The student made good use of the time allotted.

Strongly Disagree Strongly Agree

1 2 3 4 5 6 7 8 9 10

Comments:

____________________________________________________________________________________________________________________________________________________________________________________________________________________________________________________________________________________________________________________________________________________
